# Revolutionizing bone defect healing: the power of mesenchymal stem cells as seeds

**DOI:** 10.3389/fbioe.2024.1421674

**Published:** 2024-10-21

**Authors:** Yueyao Zhang, Mengke Fan, Yingze Zhang

**Affiliations:** ^1^ Trauma Emergency Center, The Third Hospital of Hebei Medical University, Shijiazhuang, China; ^2^ Key Laboratory of Biomechanics of Hebei Province, Orthopaedic Research Institution of Hebei Province, Shijiazhuang, China

**Keywords:** mesenchymal stem cells, bone defect, bone microenvironment, biomaterial, bone tissue engineering

## Abstract

Bone defects can arise from trauma or pathological factors, resulting in compromised bone integrity and the loss or absence of bone tissue. As we are all aware, repairing bone defects is a core problem in bone tissue engineering. While minor bone defects can self-repair if the periosteum remains intact and normal osteogenesis occurs, significant defects or conditions such as congenital osteogenesis imperfecta present substantial challenges to self-healing. As research on mesenchymal stem cell (MSC) advances, new fields of application have emerged; however, their application in orthopedics remains one of the most established and clinically valuable directions. This review aims to provide a comprehensive overview of the research progress regarding MSCs in the treatment of diverse bone defects. MSCs, as multipotent stem cells, offer significant advantages due to their immunomodulatory properties and ability to undergo osteogenic differentiation. The review will encompass the characteristics of MSCs within the osteogenic microenvironment and summarize the research progress of MSCs in different types of bone defects, ranging from their fundamental characteristics and animal studies to clinical applications.

## 1 Introduction

**FIGURE 1:**
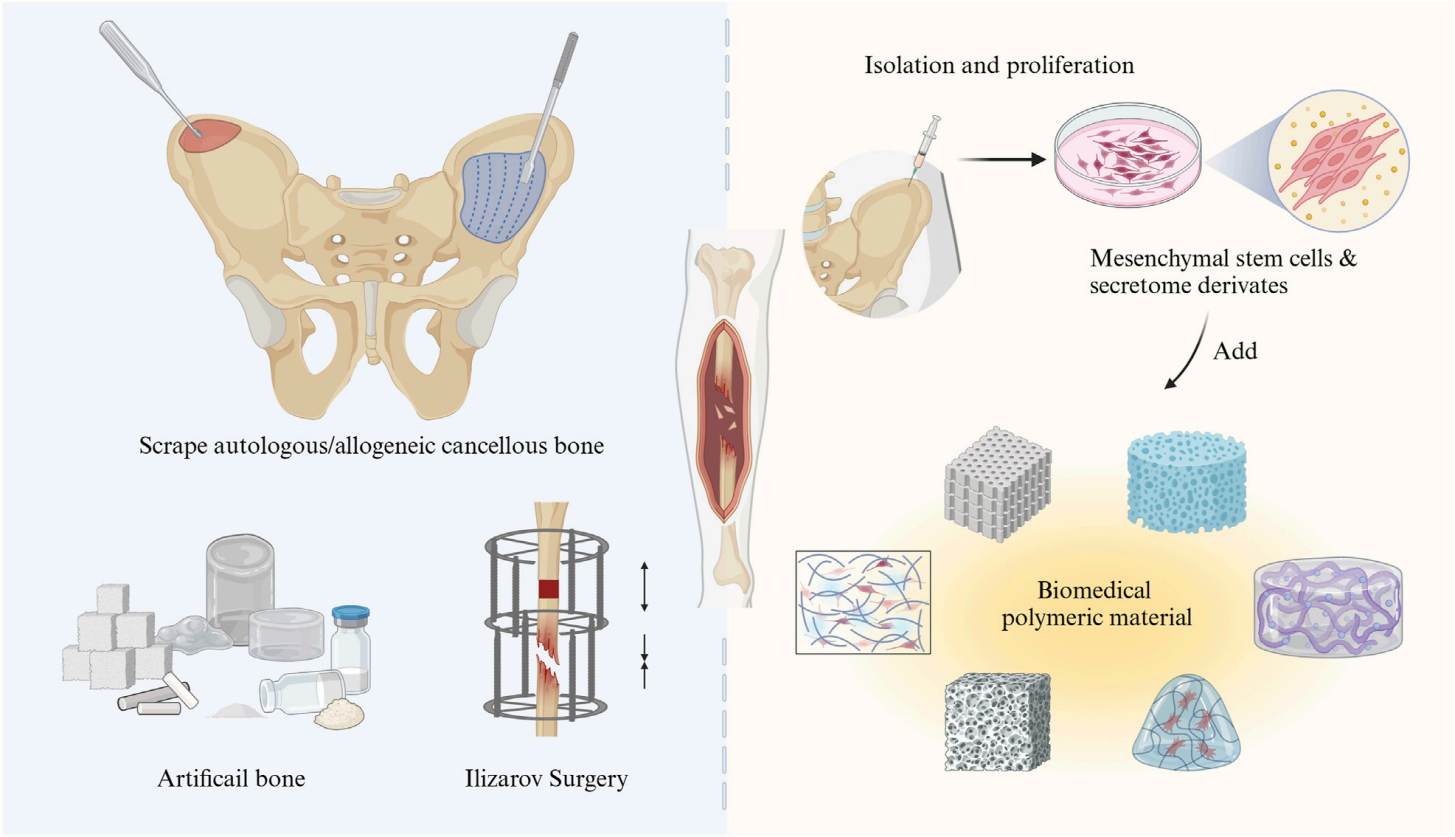
Schematic illustration of the treatment of bone tissue defects through traditional surgical strategies (left) and bone tissue engineering with MSCs (right).

With the modernization of society, skeletal injuries are becoming increasingly prevalent in advanced industrialized countries. An analysis of Global Burden of Disease (GBD) data indicates that musculoskeletal conditions are the leading contributors to disability worldwide. Bone defects are one of the important factors causing musculoskeletal conditions (Cieza et al., 2021). There are two types of bone defects: traumatic defects, which result from accidents, and pathological defects, which arise from bone diseases. When bone defects are extensive or the skeletal microenvironment is imbalanced, delayed union or nonunion would occur (Impieri et al., 2024). At present, the primary clinical methods for treating large bone defects include bone transplantation, Ilizarov technology, guided bone regeneration (GBR), and artificial bone substitute material transplantation (Semaya et al., 2016; Kim and Ku, 2020; Alford et al., 2021; Singh et al., 2021) (Figure 1). The above methods have their own advantages and disadvantages. As a primary treatment method, bone grafting with autologous or allogeneic bone can have certain effects on bone defect treatment. However, several notable limitations still exist, containing a shortage of donors, lengthy operation times, and complications (such as a high rate of bone resorption, risks of infection, and the possibility of immune rejection) during the procedure (Ferraz, 2023; Sivakumar et al., 2023). Continued research and innovation are vital to overcoming these obstacles to improve treatment status of bone defect. These challenges can also significantly impact the short-term and long-term outcome for bone defect patients.

The development of bone tissue engineering has introduced innovative ideas and strategies for the repair of tissue defects. Stem cell therapy is increasingly recognized as a promising approach in organic tissue repair, either when used alone or in combination with other treatment modalities (for instance, biomaterials and/or pharmaceuticals) (Riester et al., 2020). Mesenchymal stem cell (MSCs) are frequently utilized in both basic and clinical research due to their capacity for self-renew, repair, and differentiation into a wide range of cell types, such as osteoblasts, adipocytes, chondroblasts, neuroblasts, liver cells, and other endo- and ectodermal cells (Lee et al., 2004; Dominici et al., 2006; Bianco, 2014; Sheng, 2015). In the bone microenvironment, homing MSCs and bone marrow-derived mesenchymal stem cells (BM-MSCs) are of considerable interest in the maintenance of bone homeostasis. This interest is largely due to their key biological characteristics, including differentiation, secretion, and immunoregulation (Bruder et al., 1994; Chai et al., 2022). When a bone injury occurs, MSCs can differentiate into osteoblasts, which are the main local cells and one of the types of cells important for repairing bone damage in the bone microenvironment. Consequently, MSCs are considered ideal seeding cells for tissue engineering and regenerative medicine and have become promising candidates in the treatment of bone-related diseases. Unlike traditional surgical methods, MSC therapy leverages the regenerative potential of stem cells to culture and implant bone-like tissue *in vitro*, thereby enhancing the repair of bone defect areas and accelerating bone tissue regeneration. Furthermore, MSC therapy generally presents a lower risk of immune rejection and results in a shorter recovery time, which can reduce the incidence of complications (Aggarwal and Pittenger, 2005; Yi et al., 2022). These advantages, including enhanced biocompatibility, improved osteogenic potential, and the ability to support tissue regeneration, position MSC-based bone tissue engineering therapy as a promising and innovative treatment option for patients with bone defects or injuries (Figure 1). A comprehensive overview of the latest advancements and realistic applications of MSCs is therefore essential. This paper summarizes the use of MSCs in bone defect therapy by dividing the discussion into two parts: the fundamental roles and mechanisms of MSCs and their related applications in both basic and clinical research. Finally, the application prospects and future research directions for MSCs are explored.

## 2 Basic characteristics and cell markers of MSCs

MSCs have traditionally been considered as multipotent stem cells characterized by a spindle-shaped morphology and adherent properties (Dominici et al., 2006). They were discovered during bone marrow transplantation (Friedenstein et al., 1968) and are known to express specific surface antigens, including CD73 (with a positive rate >95%), CD90, and CD105, and to lack expression of hematopoietic cell-associated surface antigens (including CD45, CD34, CD11 or CD14, CD79a or CD19, and HLA-DR), in accordance with the identification criteria proposed by the International Society for Cell Therapy (ISCT) (Dominici et al., 2006).

Given the absence of major histocompatibility complex (MHC), Fas ligand (apoptosis-mediating surface antigen Fas) and T-cell costimulatory molecules, MSCs are not easily recognized by immune cells (Najar et al., 2012; Oh et al., 2022). Studies have found that human MSCs typically express low levels of MHC-I molecules and do not express MHC-II molecules or costimulatory molecules (such as CD86, CD80, CD40, and CD40L), which makes them excellent candidates for cell therapy (Chamberlain et al., 2007; Lechanteur et al., 2016; Keshavarz Shahbaz et al., 2022). Recent research further stated that MSCs enable the evasion of natural killer (NK) cell-mediated cytotoxicity and exert immunosuppressive activity with low levels of MHC-I molecules (Oh et al., 2022). However, in the presence of pro-inflammatory cytokines, such as interferon-gamma (IFN-γ), MHC-II molecules will be upregulated on MSCs (Cassano et al., 2018). MHC-II molecules are mainly involved in presenting antigens to helper T cells, which are essential for coordinating immune responses (Maizels and Withers, 2014). This upregulation of MHC-II allows MSCs to effectively present antigens to helper T cells and activate immune responses when necessary (Omoto et al., 2014). Studies have demonstrated that the intratumoral implantation of MSCs, combined with peripheral IFN-γ immunotherapy can cure glioma-bearing rats (Ströjby et al., 2014). Moreover, MSCs also exhibit immune rejection during allograft when MHC-I and MHC-II of the recipient mice are mismatched (Eliopoulos et al., 2005). Considering these points, the immune interactions of MSCs are complex and dependent on various factors. However, the unique MHC expression profile, characterized by low levels of MHC-I and inducible MHC-II expression, endows them with immunomodulatory properties that can be harnessed for therapeutic purposes.

MSCs can be isolated from many tissues, such as bone marrow, adipose tissue, muscle, synovial fluid (Alhadlaq and Mao, 2004), umbilical cord, chorion, placenta, amnion (Asgari et al., 2017), dental pulp, or gingiva (Paganelli et al., 2021). At present, BM-MSCs, adipose-derived mesenchymal stem cells (AD-MSCs), umbilical cord mesenchymal stem cells (UC-MSCs), amniotic membrane mesenchymal stem cells (AM-MSCs), and placental mesenchymal stem cells (PMSCs) are commonly used in the study of bone injury therapy. Nonetheless, MSC-based therapies encounter several challenges, including limited self-renewal capacities, variability in the availability of donor-derived MSCs, and the financial implications associated with donor screening processes (Zhou et al., 2021). As an alternative approach, MSCs derived from induced pluripotent stem cells (iPSCs), which offer the potential for more standardized cell preparations. MSCs have emerged as a particularly advantageous source of iPSCs due to their ease of isolation and cultivation, as well as their relatively straightforward reprogramming compared to terminally differentiated cells (Shao et al., 2013).Therefore, it is crucial to identify the commonalities and differences between various MSCs types in the treatment of bone defects.

Studies have shown that the different molecular antigens expressed on MSCs can be used to distinguish and identify their source and function (Table 1). The more commonly used BM-MSCs highly express numerous surface markers, such as CD10, CD13, CD29, CD44, CD49c, CD49f, CD59, CD73, CD81, CD90, CD105, CD106, CD143, CD146, CD147, CD151, CD166, CD271, CD276, low-affinity nerve growth factor receptor (LNGFR), podocalyxin-like protein 1 (PODXL), Stro-1, and bone morphogenetic protein receptor type 1A (BMPR-1A), with little or no expression of CD3, CD11b, CD14, CD19, CD34, CD36, CD45, CD79a, CD117, and CD133 (Jones et al., 2002; Shiota et al., 2007; Pachón-Peña et al., 2011; Amati et al., 2018). Nevertheless, significant differences in the percentage of surface expression were observed among MSCs from different sources. Compared with BM-MSCs, AD-MSCs showed surface positives for CD34 and CD36, while showing only weak positivity for CD49f, CD106 and PODXL (Pachón-Peña et al., 2011). More importantly, CD143 is specifically expressed on BM-MSCs and is restricted to only adult sources (Amati et al., 2018). Additionally, CD106 serves as a marker of placental chorionic plate-derived mesenchymal stem cells (CP-MSCs), which possess strong immune regulation functions (Fukiage et al., 2008; Yang et al., 2013). Moreover, CD271 has been identified as a crucial marker during the high-purity isolation of BM-MSC, as CD271^high^ BM-MSCs contain colony-forming units-fibroblast (CFU-F) and other positive markers, including CD10, CD13, CD73, and CD105 (Bühring et al., 2007).

**TABLE 1:** Common positive and negative markers of MSCs and their significance.

MSCs positive markers and their characters		
Source	Markers	Characters
Universal Markers	CD73 (Ode et al., 2013)	Catalyzing adenosine shape from AMP and further promoting osteogenesis activity
	CD90 (Yang et al., 2015; Picke et al., 2018)	Promoting bone formation and the interaction among cells
	CD105 (Clarkin et al., 2013; Fan et al., 2016)	Promoting chondrogenesis, and vascular homeostasis via TGF-β
BM-MSCs	CD271 (Bühring et al., 2007; Mifune et al., 2013)	Greater expansion potential and elevated expression levels of chondrogenic genes
CD-MSCs	CD106 (Fukiage et al., 2008; Yang et al., 2013)	More effective in modulating T helper subsets and expressing a greater variety of cytokines, with enhanced immunosuppressive activity

MSCs negative markers and their expression cells	
CD3	T cell
CD11b	Monocyte-macrophage
CD14	Monocyte-macrophage
CD19	B cell
CD34	Primitive hematopoietic cells; endothelial cell
CD36	Monocyte; platelet; immature red blood cell; plasmacytoid dendritic cell
CD45	Immunocyte
CD79a	B cell
CD117	Primitive myeloid cell; mast cell
CD133	Tumor stem cell; hematopoietic stem cell; neural stem cells; embryonic stem cell

In addition to expressing specific markers, the cytokines secreted by different types of MSCs are also key in distinguishing MSC clusters. For instance, BM-MSCs, AD-MSCs, and UC-MSCs secrete varying levels of hepatocyte growth factor (HGF), transforming growth factor-β1 (TGF-β1), interleukin-6 (IL-6), interleukin-10 (IL-10), and prostaglandin E2 (PGE2) (Chen et al., 2015; Nakao et al., 2019). Granulocyte colony-stimulating factor (G-CSF) and granulocyte-macrophage colony-stimulating factor (GM-CSF) are predominantly produced by BM-MSCs, whereas vascular endothelial growth factor (VEGF) expression in BM-MSCs is relatively low (Ostanin et al., 2011; Dabrowski et al., 2017; Tyrina et al., 2023). A significantly higher 26S proteasome activity was detected in AD-MSCs than in BM-MSCs. Levels of intercellular cell adhesion molecule-1 (ICAM-1), integrin α5 and integrin α6 were significantly higher in AD-MSCs compared to BM-MSCs (Abu-El-Rub et al., 2024). Compared with BM-MSCs, AD-MSCs secrete higher levels of Th1/pro-inflammatory factors, such as IFN-γ, interleukin-2 (IL-2), interleukin-1β (IL-1β), and tumor necrosis factor-alpha (TNF-α). PMSCs produce higher levels of Th2/anti-inflammatory factors, such as interleukin-4 (IL-4), IL-6, and monocyte chemoattractant protein-1 (MCP-1), compared to BM-MSCs (Ostanin et al., 2011). These findings indicate that MSCs from different sources possess distinct immunomodulatory and adhesion profiles, which may significantly impact their roles in tissue repair and regeneration. Moreover, embryonic tissue-derived MSCs secrete low levels of matrix metalloproteinases (MMPs) (Bian et al., 2022) (Table 2). In pathological environments, cytokine secretion will change tremendously. For instance, the secretion of cytokines from BM-MSCs will increase after stimulation with high concentrations of lipopolysaccharide or endotoxin (Ostanin et al., 2011). Recent research has emphasized an even more nuanced aspect of MSC function: the release of extracellular vesicles (EVs). These nanoscale vesicles carry a cargo of bioactive molecules such as proteins, lipids, and RNAs that can influence recipient cells. EVs derived from BM-MSCs are enriched in anti-inflammatory proteins compared to EVs from other sources, which are associated with less organ damage (Blanco et al., 2023). Additionally, MSCs from different sources showed different differentiation capabilities. For example, muscle-derived MSCs regularly failed to form any histologically identifiable bone. In contrast, periosteum- and cord blood-derived MSCs can form bone, but they do not establish a hematopoietic microenvironment. BM-MSCs, however, are capable of forming bone and establishing the hematopoietic microenvironment (Sacchetti et al., 2016). Human BM-MSCs demonstrate the highest bone regenerative potential compared to those derived from adipose tissue, umbilical cord, mature chondrocytes, and skin fibroblasts. BMSCs were able to form cartilage discs *in vitro* and fully regenerate critical-size femoral defects mice. The binding sites of enhancers and promoter regions of ossification-related genes, which are commonly expressed transcription factors, are exclusively available in BM-MSCs. And the secretion of osteopontin initiates the healing of defects and the formation of bone hard calluses (Hochmann et al., 2023). These results suggest that MSCs with specific markers, distinct sources, and varying secretory factors can serve as multiple carriers for research and treatment, aiding scholars in constructing therapeutic complexes.

**TABLE 2:** Types and significance of common paracrine factors in MSCs.

Source	Common cytokines	Function	Reference
BM-MSCs	TGF-β1	Inhibit macrophage activation; reduce the secretion of pro-inflammatory factors; downregulate immune response	Wahl et al. (2000)
	PGE_2_	Anti-inflammatory response; enhance the therapeutic effect of MSCs; induce mono-macrophage differentiation toward M2	Németh et al. (2009), Gomez et al. (2013), Kota et al. (2017)
	IL-8	Anti-inflammatory response; induce mono-macrophage differentiation toward M2	Li et al. (2019b)
	VEGF	Promote angiogenesis	Du et al. (2016)
AD-MSCs	HGF	Inhibit the function of dendritic cells; inhibit the activation of T cells; inhibit inflammatory response; mediate the functional recovery of MSCs	Ito et al. (2005), Benkhoucha et al. (2010), Bai et al. (2012)
	IL-1β	Anti-inflammatory response; induce mono-macrophage differentiation toward M2; promote T-reg cell activation, inhibit the activation of Th-1, Th-17 cell	Harrell et al. (2020)
	IL-2	Mediate T cell proliferation and activation	Park et al. (2011)
	IL-10	Anti-inflammatory response, inhibit T cell proliferation, induce monocyte-macrophage differentiation to M2	Yang et al. (2009), Deng et al. (2012)
	IFN-γ	Inhibit T cell proliferation and downregulate immune response	Kim et al. (2018)
	TNF-α	Regulate inflammation and induce monocyte-macrophage differentiation to M1. Some studies suggest that it increases exosome secretion, monocyte-macrophage differentiation to M2, and inhibits bone resorption	Murray (2017), Nakao et al. (2021)
	GM-CSF	Regulate immune response and promote cell mobilization, proliferation, and migration	Waghray et al. (2016), Kim et al. (2019)
UC-MSCs	IL-6	Anti-inflammatory reaction; induce monocyte-macrophage differentiation into M2	Bernardo and Fibbe (2013), Li et al. (2013)
PMSCs	G-CSF	Modulate immune response and mediate injury repair	Sasaki et al. (2017), Avila-Portillo et al. (2020)
	IL-4	Anti-inflammatory reaction; induce monocyte-macrophage differentiation into M2	Muñoz et al. (2020)
	CCL2	Recruit macrophages; reduce inflammatory response; promote injury repair	Whelan et al. (2020)
	IGF-1	Anti-inflammatory response; promote tissue repair	Ikeda et al. (2017), Dn et al. (2018)

## 3 Origins and immunomodulatory role of MSCs in bone injury or healing

**FIGURE 2:**
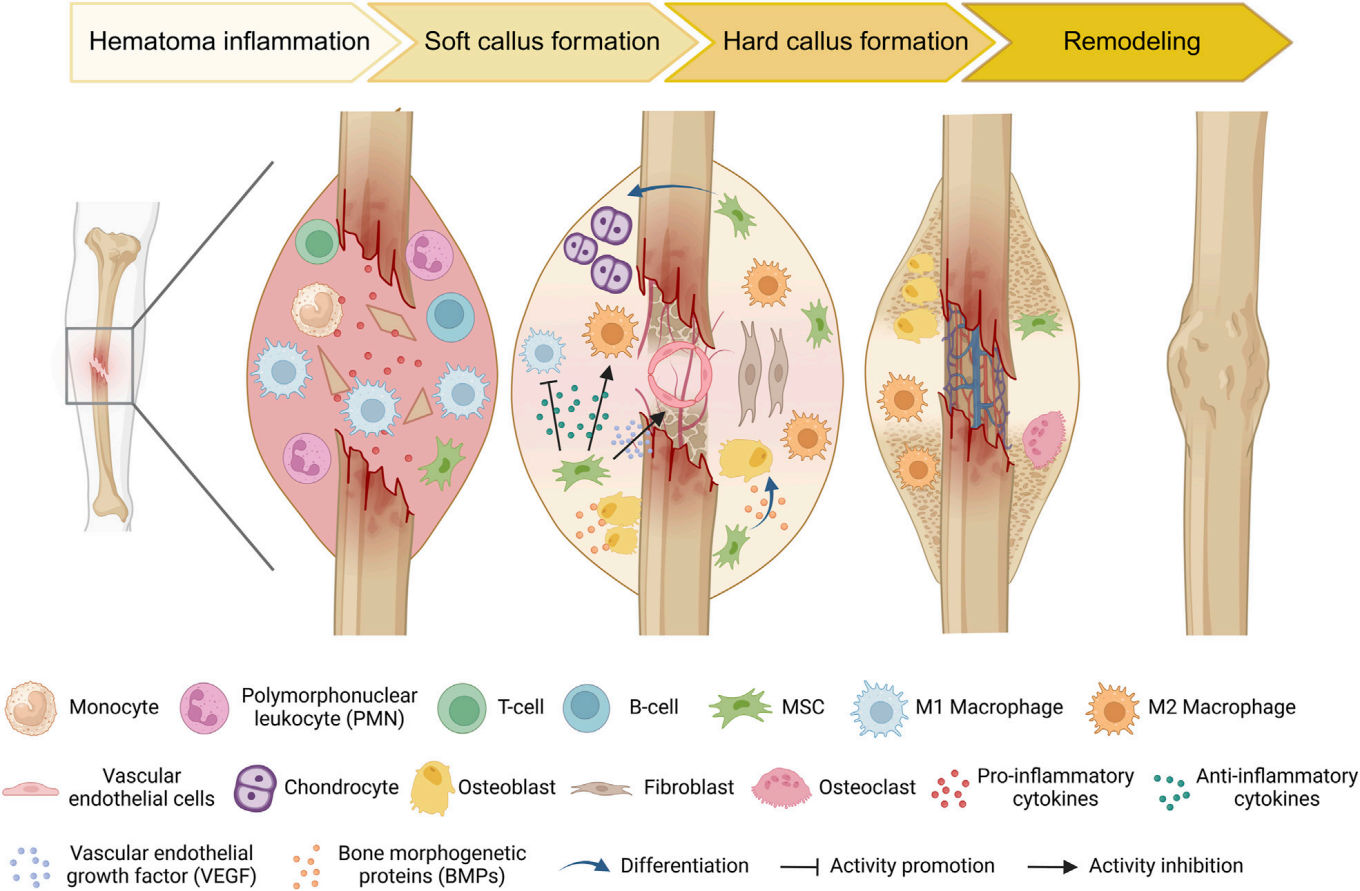
Schematic diagram of the typical natural bone healing process and cell dynamic transformation. During the inflammatory stage, injury triggers the recognition of damage-associated molecular patterns (DAMPs) by innate immune cells. These cells then secrete pro-inflammatory cytokines to recruit additional inflammatory cells and initiate an adaptive immune response. Concurrently, MSCs are recruited to the injury site. As necrotic debris is cleared and the immunomodulatory effects of MSCs, anti-inflammatory cells (e.g., M2 macrophages) increase, which promotes the differentiation of MSCs into osteoblasts and chondrocytes, thereby accelerating bone matrix mineralization and callus formation. As new bone tissue forms, the fracture site becomes more stable. Subsequently, osteoclasts begin the remodeling process by resorbing old and loose bone tissue, ultimately restoring bone shape and strength.

The healing process following bone injury involves a complex interaction between various cells and cytokines, with MSCs playing a crucial regulatory and reparative role (Figure 2). During the bone repair process, the recruitment of circulating stem/progenitor cells to the site of injury occurs as a normal biological response (Peihong et al., 2018). The ability of circulating or administered MSCs to migrate and integrate into the bone tissue environment is referred to as MSC homing (Ullah et al., 2019). The homing process is the initial step of bone formation, enabling MSCs to effectively migrate to the area of the bone defect, where they can repair and regenerate tissue as needed (Wang et al., 2013). This is an important factor in bone regeneration, as it indirectly reflects the ability of local bone to regenerate by recruiting progenitor cells (Liesveld et al., 2020; Guo et al., 2024). In the bone microenvironment, MSCs can originate from peripheral stromal vessel walls on the trabecular bone surface or in interfibrillar spaces (da Silva Meirelles et al., 2009). MSCs can differentiate into bone-related cells, such as osteoblasts and chondrocytes. Intravenous injection of CD271-selected MSCs within 24 h of a mouse femur fracture resulted in significant accumulation at the fracture sites for at least 7 days (Dreger et al., 2014). With technical advancements, research on MSCs homing has made substantial progress. Cytokines and chemokines, such as stromal cell-derived factor 1(SDF-1) and its receptor chemokine receptor type 4 (CXCR4) play important roles in maintaining mobilization, trafficking and homing of stem cells from bone marrow to the site of injury (Ye et al., 2020). After injury, such as fractures, the local hypoxic environment stimulates the secretion of these cytokines and chemokines, leading to the mobilization of MSCs. Lauer et al. reported that the application of SDF can promote MSCs homing to the fracture site, which could be conducive to fracture healing (Lauer et al., 2020). Furthermore, the upregulation of the chemokine C-X-C motif ligand 12 (CXCL12) at the bone injury site could promote the migration ability of MSCs expressing CXCR4 (Yellowley, 2013).The widely employed short-wave therapy in clinical practice enhances MSCs homing during fracture healing by upregulating the expression of cytokines such as SDF-1 and CXCR-4 in the callus (Ye et al., 2020). MSC homing marks the initiation of bone formation. Improving the migratory capacity of MSCs holds immense importance for the process of bone formation.

In the early stages of bone injury, cells in the bone microenvironment are damaged. Due to the release of injury-associated cytokines, neutrophils, monocytes, and lymphocytes successively infiltrate into the injury site (Chow et al., 2022). At this stage, homing MSCs mediates necrotic tissue clearance and bone regeneration through immune regulation. For example, during the early phases of bone infection, tissue-specific MSCs induce increased recruitment, activation, and sensitization of neutrophil granulocytes (Brandau et al., 2010). In the bone microenvironment, the level of type I interferon (IFN-I) will increase once MSCs are stimulated by injury cytokines. The IFN-I produced by MSCs can also enhance the effector function of NK cells in the early stage of bone repair (Petri et al., 2017). In addition, several studies have shown that as a type of multipotent stem cell, MSCs play an important role in the regulation of osteoimmunology. Bartholomew et al. was the first to discover the immunosuppressive ability of MSCs by demonstrating that MSCs inhibit lymphocyte proliferation *in vitro* and allogeneic skin transplantation rejection *in vivo*, thus confirming that MSCs have an immunoregulatory effect in bone (Bartholomew et al., 2002). In subsequent studies, it was found that MSCs could exercise immunomodulatory effects in both innate and adaptive ways (Glennie et al., 2005; Pan et al., 2024). Therefore, MSCs can serve as promising targets for early intervention therapies in tissue regeneration.

In the process of bone regeneration, the immunomodulatory properties of MSCs would be directly conducive to osteogenesis in inflammation conditions. MSCs regulate immune responses in the skeletal system via juxtacrine and paracrine signaling (Harrell et al., 2021). Preconditioning MSCs with pro-inflammatory cytokines alters their secretory profile and osteogenic potential. Ren and colleagues discovered that the immunosuppressive properties of MSCs are induced by IFN-γ, TNF-α, interleukin-1 alpha (IL-1α), and IL-1β (Ren et al., 2008). *In vitro* simulation of early pro-inflammatory conditions in fracture healing reveals that CD146-positive MSCs exhibit strong immunomodulatory and pro-angiogenic activities (Herrmann et al., 2019). Furthermore, the investigation reveals that the immunosuppressive function of MSCs is mainly mediated through the secretion of cytotoxic T lymphocyte antigen 4 (CTLA-4), especially under hypoxic conditions (Gaber et al., 2018). This immunomodulatory property can regulate local inflammation, creating a more favorable environment for bone healing and promoting the osteogenic differentiation of MSCs. Among the various elements influencing this process, the interaction between MSCs and macrophages is essential for maintaining bone homeostasis and facilitating regeneration (Hu et al., 2023). MSCs enhance healing by secreting a variety of cytokines and chemokines, such as VEGF-α, Insulin-like growth factor 1 (IGF-1), epidermal growth factor (EGF), keratinocyte growth factor, angiopoietin-1, SDF-1, macrophage inflammatory protein-1 (MIP-1), that promote the migration and proliferation of macrophages and endothelial cells (Chen et al., 2008). High IFN-γ and TNF-α levels could stimulate MSCs to secrete a large number of immunosuppressive cytokines, such as HGF, TGF-β, indoleamine 2,3-dioxygenase (IDO), PGE2, and nitrous oxide (NO) (Hackel et al., 2021). In addition, the specific C-C motif chemokine ligands 2 (CCL2) released from MSCs may facilitate their interaction with macrophages in the skeletal system (Shen et al., 2016; Toya et al., 2023). When MSCs are co-cultured with macrophages, the pro-inflammatory cytokines TNF-α, IL-1β, and IL-6 from the macrophages are suppressed while the anti-inflammatory cytokine IL-10 is produced (Hu et al., 2015; Chen et al., 2019). A study conducted by Heo et al. demonstrated that MSCs could induce macrophages to transit into the M2 phenotype through the action of exosomes *in vitro* (Heo et al., 2019)*.* Mesenchymal stem cell-conditioned media (MSC-CM), particularly when stimulated with IL-4, exhibited anti-inflammatory effects by inhibiting the activation of lipopolysaccharide (LPS)-stimulated macrophages, downregulating inflammatory mediators, and suppressing key inflammatory pathways (Jin et al., 2022). Moreover, TNF-stimulated gene 6 protein (TSG-6), IL-6, and IL-10 from MSCs to macrophages exert similar effects as mentioned above (Lo Sicco et al., 2017; Arabpour et al., 2021). M2 macrophages could further enhance the proliferation of CD4^+^CD25^+^FOXP3^+^ T cells (Tregs), a subtype of immunosuppressive T cell (Tiemessen et al., 2007; Zhao W. et al., 2020). Correspondingly, the enhancement of M2 macrophages promotes the osteogenic differentiation of MSCs (Mahon et al., 2020). Additionally, the paracrine interactions between MSCs and macrophages are regulated by 1,25-dihydroxyvitamin D3 (Saldaña et al., 2017). Further experiments showed that macrophage mitochondrial transfer can promote the osteogenic differentiation of MSCs. Increased mitochondrial transfer of M1-like macrophages to MSCs triggers a reactive oxygen species (ROS) burst, which leads to metabolic remodeling (Cai et al., 2023; Qiu et al., 2024). In summary, the combined effects of MSCs on immune cells regulate the inflammatory response, particularly through the interplay and communication mechanisms between MSCs and macrophages, which contribute to the establishment of a more stable immune microenvironment, stimulate angiogenesis, and promote the proliferation and differentiation of osteoblasts.

**FIGURE 3:**
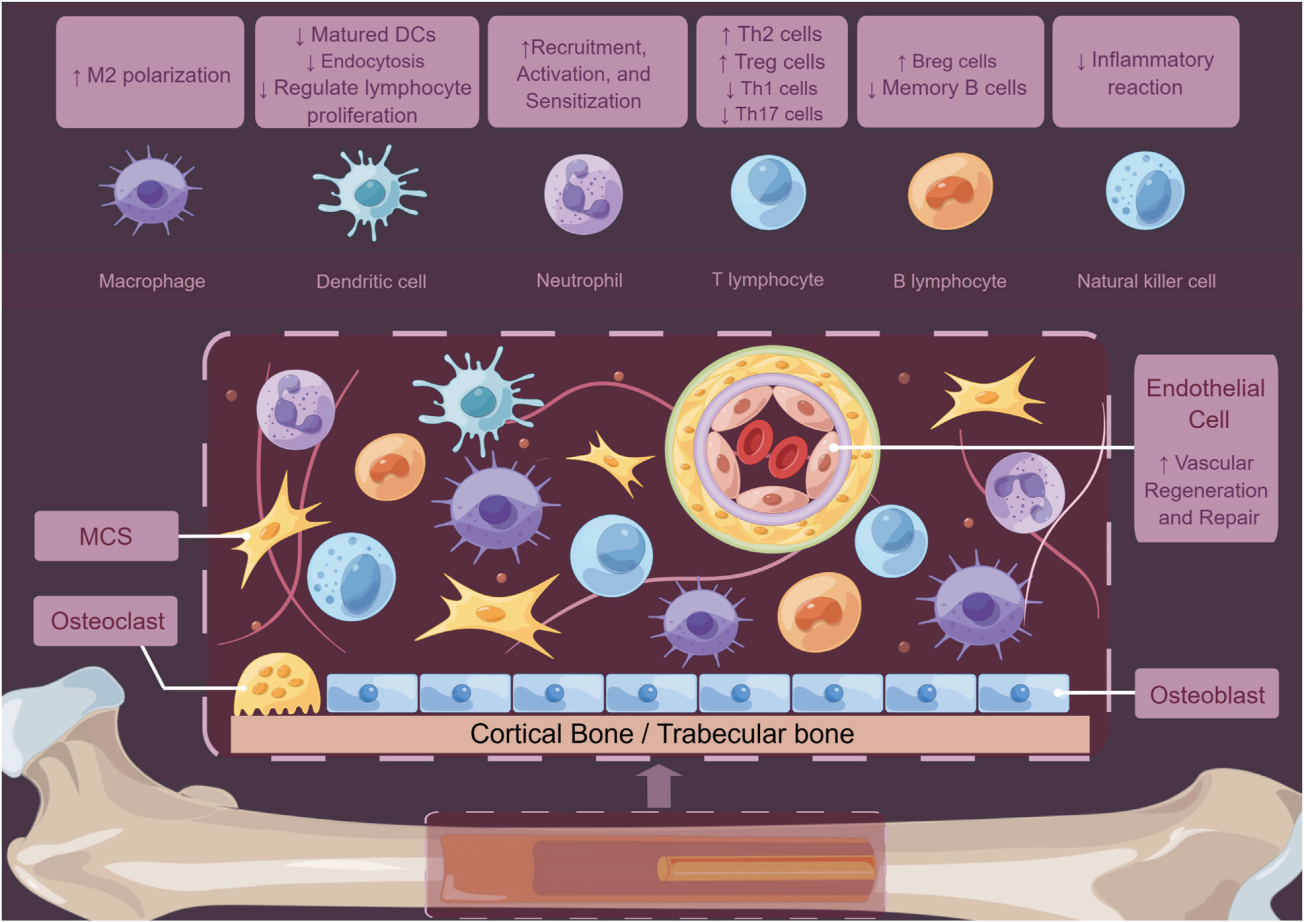
Schematic diagram of how MSCs regulate the bone marrow microenvironment. MSCs not only act on immune cells such as macrophages, DCs, neutrophils, T cells, B cells, and NK cells to play crucial immunomodulatory roles but also regulate tissue cell functions and mediate vascular repair and regeneration through autocrine and paracrine proteins.

Except for macrophages, MSCs can modulate local immune state indirectly through interactions with other immune cells. For instance, MSCs can induce the formation of Tregs via heme oxygenase-1 (HO-1) and IL-10. Scholars found that MSCs promote the proliferation of Th2 cells and Tregs by inhibiting Th1 cells, Th17 cells, and B cells (Schena et al., 2010; Fan et al., 2012; Luz-Crawford et al., 2013). Tregs are known to positively impact bone regeneration by stimulating the osteogenic differentiation of MSCs (Li et al., 2024). Moreover, the CD146^high^ MSCs play an inhibitory role in host T cells, which is related to apoptosis of T cells (Bikorimana et al., 2022). In the regulation of dendritic cell (DC), both MSCs and their supernatants could decrease DC endocytosis by impairing IL-12 levels (Zhang et al., 2004). MSCs could elevate the population of CTLA-4^+^ DCs, which play a crucial role in inducing naïve T cells, mainly polarizing them into IL-10^+^IL-17^+^ T helper cells (Mázló et al., 2021). Exosomes derived from MSCs can also promote the maturation of DCs by reducing IL-6 levels while increasing IL-10 and TGF-β levels. This process enhances antigen presentation, initiates the immune response, and coordinates the repair process (Shahir et al., 2020).The MSCs could also increase the levels of CD24^high^CD38^high^ B cells and IL-10-producing B cells while reducing the number of memory B-cells (Carreras-Planella et al., 2019) (Figure 3). Based on the findings of current studies, MSCs primarily exhibit anti-inflammatory properties and promote bone regeneration at defect sites by modulating their cytokines expression in the bone microenvironment. This suggests that MSCs can be multipotent seeds for bone tissue engineering. Given their numerous immunomodulatory effects, MSCs are crucial not only for the repair of bone defects but also for the management of various bone-related diseases, including osteoarthritis. MSCs can suppress the release of inflammatory mediators, attenuate the activation of macrophages, T and B cells, and facilitate cartilage regeneration and repair. These mechanisms collectively contribute to a reduction in inflammation and tissue damage, thereby enhancing joint function (Fahy et al., 2014; Fichadiya et al., 2016; Hu et al., 2018; Labinsky et al., 2020; Zhao X. et al., 2020; Zhu et al., 2020). MSCs play a crucial role in maintaining the homeostasis and equilibrium of the bone microenvironment (Lin et al., 2021; Shen and Shi, 2021). These findings open new ways for treatment options in bone regeneration processes. The potential for designing various types of MSC carriers to meet the requirements for bone defect healing within the skeletal system remains a significant area of exploration.

## 4 MSCs in bone defect healing

**FIGURE 4:**
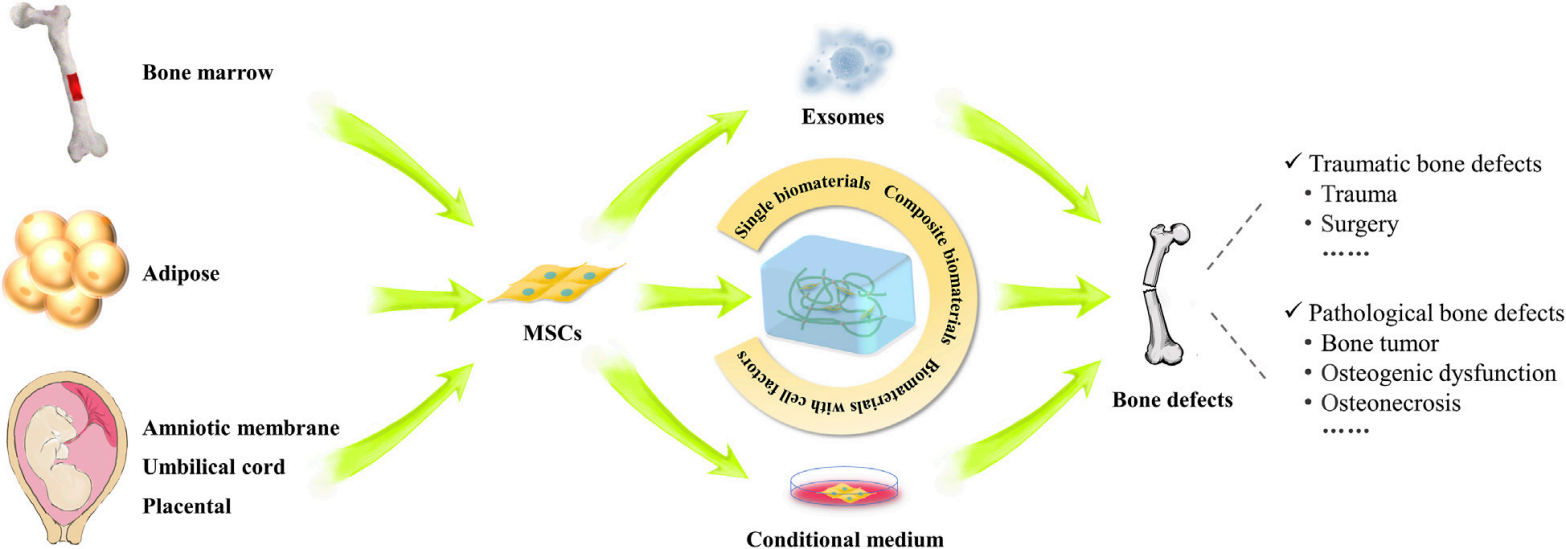
Study and applications of MSCs in the repair of various bone defects. The schematogram illustrates the use of MSCs from different sources in the treatment of bone defects. MSC implantation, conditioned medium, exosomes and MSC composite biomaterials can promote the repair of various types of bone defects.

Currently, MSCs have been used as therapeutic agents for a variety of disorders, particularly in bone regeneration, due to their capacity for self-renewal, migration, and immunomodulation (Song et al., 2020). Bone defects can generally be classified into two categories: traumatic and pathological. When bone defects occur, the release of inflammatory factors will attract MSCs to migrate into the injury sites. Subsequently, MSCs secrete cytokines locally to facilitate signaling communication essential for bone formation (Kang et al., 2022). Although significant advancements have been made in the treatment of bone defects within clinical orthopedics, large-scale bone defects--defined as those greater than 1–2 cm with a more than 50% loss in bone circumference--continue to pose a clinical challenge (Schemitsch, 2017). Thus, repairing large defects has been a high-interest topic in recent years. Notably, MSCs are regarded as ideal candidates for assisting in the repair of bone defects. Numerous studies have demonstrated that the combined application of MSCs with cell-secreted substances, such as exosomes and cytokines, along with genetic modification and preconditioning, could help to mitigate the limitations of these methods within the bone microenvironment (Huang et al., 2020; Pawitan et al., 2020; Nakao et al., 2021; Zou et al., 2023). The combination of bioactive materials and MSCs may further enhance osteogenic effect at bone defect sites (Niu et al., 2017; Xiongfa et al., 2020; Xie et al., 2022; Park et al., 2024). As of January 2024, over 70 clinical trials involving the use of MSCs in bone regeneration, bone tumors, osteogenic dysfunction and osteonecrosis have been carried out worldwide (https://trialsearch.who.int and http://www.clinicaltrials.gov, accessed by 30 January 2024) (Figure 4).

### 4.1 MSCs in traumatic bone defects

Traumatic fractures and surgical osteotomies are common types of bone defects. In general, traumatic fracture repair can be divided into four phases: the initial inflammatory response, soft callus formation, hard callus formation, initial bony union and bone remodeling (Schindeler et al., 2008) (Figure 2). If patients present with a local or systemic infection, poor alignment, multiple combined injuries, or are in poor physical condition, delayed or nonunion of bone will occur (Hak et al., 2014; Tian et al., 2020). Thus far, there are many ways to efficiently resolve the above concerns. Various therapeutic strategies, including autologous bone grafting, allogeneic bone grafting, and the application of synthetic bioactive materials, have demonstrated substantial clinical potential for addressing bone defects. Despite these advancements, several limitations remain that impact their efficacy and applicability (Ferraz, 2023). In response to these challenges, the field of MSCs research has become an emerging trend. The focus of bone defect treatment has evolved from merely achieving bone continuity to employing bioengineering approaches aimed at optimizing the osteo-microenvironment.

MSCs can differentiate into osteoblasts to initiate local bone repair or secrete bioactive substances indirectly influence bone regeneration (Asgharzadeh et al., 2018). Huang et al. used femoral fracture models of mice to demonstrate that both systemic and local application of MSCs promoted fracture healing equally through direct differentiation into osteoblasts (Huang et al., 2015). Above all, scholars found that compared with MSCs, MSC-CM had higher concentrations of cytokines, which could accelerate bone healing in bone defect sites with cellular secretions (Osugi et al., 2012). The CM secreted by MSCs primarily consists of numerous active substances, including cytokines, immune factors, and other components (Al-Sharabi et al., 2023). MSC-CM enhances macrophage migration and induces the switching of macrophages from M1 to M2 types at an early stage of osteogenesis (Katagiri et al., 2022). Benavides-Castellanos et al. concluded that the application of MSC-CM in bone defects could have a beneficial effect on the repair and regeneration of bone tissue, which shows that paracrine effects play a crucial role in MSC treatment (Benavides-Castellanos et al., 2020). To further investigate the reason for this observation, scholars found that the exosomes in MSC-CM are critical effector substances in osteogenesis and angiogenesis (Hu et al., 2015; Furuta et al., 2016; Diomede et al., 2018). As the research progresses, exosomes derived from stem cells have been extensively studied for their role in promoting regeneration and reconstruction of various tissues as “cell-free” therapies. Li et al. (2022) used MSCs as mediators that were genetically engineered with the bone morphogenetic protein-2 (BMP2) gene to produce exosomes (MSC-BMP2-Exo), which have potential applications in the process of bone repair. The engineered MSCs function as cellular factories, and produced exosomes exhibit excellent biocompatibility and homing ability. Other than enhancing osteogenic differentiation, MSC-Exos increase the expression of VEGF and hypoxia inducible factor-1α (HIF-1α), which are crucial for promoting angiogenesis and accelerating fracture healing (Zhang et al., 2019). Liu et al. (2020) found that the hypoxic pretreatment of MSCs enhances the production of exosome miR-126 by activating HIF-1a, which mediates the SPRED1/Ras/Erk signaling pathway to promote fracture healing. In addition to directly regulating bone regeneration, MSCs can be systemically infused to reduce inflammation. Liu et al. found that inflammatory cytokines (IFN-γ and TNF-α) at implantation sites are downregulated by systemic infusion of MSCs, which upregulates Tregs (Liu et al., 2015). This approach can also enhance bone regeneration in MSC-seeded scaffolds. The results of the meta-analysis strengthen the evidence supporting the systemic application of MSCs in bone regeneration (Fu et al., 2021). However, detailed mechanisms should be explored in future studies. The diverse capabilities of MSCs, including direct osteogenic differentiation, paracrine effects through MSC-CM, and the use of engineered exosomes, underscore their significant potential for promoting bone regeneration and accelerating fracture healing. Their ability to enhance bone regeneration both directly and indirectly makes them ideal biotherapeutic agents.

With the development of osteogenesis-promoting biomaterials, the combination of biomaterials and MSCs has been precisely advanced to treat bone defects with suitable biocompatibility, physicochemical stability, and safety. In general, MSCs are seeded on scaffolds, which are then transplanted to bone defects for bone regeneration. Currently, delivering MSCs via biodegradable scaffolds is an important direction in the study of bone repair. Such bioactive scaffolds not only enhance the adhesion, growth, and survival of MSCs but also guide MSCs to differentiate into osteoconductive structures (Venkataiah et al., 2021). Marcacci and colleagues first reported the use of autologous MSCs expanded *in vitro* and inoculated onto a porous ceramic scaffold of hydroxyapatite (HA), a main inorganic component of bone (Marcacci et al., 2007). Wang et al. transplanted coral scaffolds with rabbit-derived AD-MSCs into bone defects in nude mice, and the rate of bone formation was significantly enhanced after 8 weeks (Wang et al., 2021). Moreover, bone defect healing can be improved by using nanomaterials to affect the polarization of macrophages and their interaction with MSCs. M1 macrophages initiate and maintain an inflammatory response in the early stages of bone healing to help remove damaged tissue; M2 macrophages promote the formation and remodeling of new bone in the middle and late stages of bone healing (Murayama et al., 2024). HA and poly (methyl methacrylate) (PMMA) particles could drive the polarization of macrophages towards an inflammatory phenotype, leading to an inflammatory cascade which culminates in periprosthetic osteolysis. Pre-treatment of macrophages with pharmacological inhibitors of these molecules in turn prevents macrophage polarization and dampens inflammatory cytokine production (Mahon et al., 2018). With the improvement of technology, according to current research reports, nano hydroxyapatite particles (BMnP) polarize human macrophages towards M2 phenotype, and further enhance MSC osteogenesis in an IL-10-dependent manner (Mahon et al., 2020). The GAD/Ag-pIO scaffold enhances osteogenic differentiation and fracture healing through immunomodulation and promotion of macrophage mitochondrial transfer (Qiu et al., 2024). Fibrin-MSC composites triggered rapid attraction of host cells into the hydrogel, forming a migration front dominated by M1 macrophages and endothelial progenitor cells, which stimulates early tissue maturation and primitive blood vessel formation, thereby promoting long bone healing in rats (Seebach et al., 2014). IL-4 overexpressing MSCs within macroporous gelatin-based microribbon (μRB) scaffold promoted the expression of M2 markers and increased the chemotaxis of macrophages into the scaffold, which contributes to MSC osteogenesis differentiation and helps bridge the long bone defect (Ueno et al., 2020).

To better improve the effectiveness of combination strategies, different kinds of active ingredients have been applied in bone defect treatment with a collaborative therapeutic effect. Xie et al. (2016) found that hydroxyapatite/collagen/chitosan (HAp/Col/CTS) could significantly promote the adhesion, proliferation, and osteogenesis of MSCs. When a 30% HAp/CTS scaffold combined with MSCs from second trimester human amniotic fluid was implanted into the bone defect in a rabbit tibia, the bone healing rate improved significantly (Mohammed et al., 2019). Kamali et al. prepared a gelatin/nanohydroxyapatite (G/nHAp) scaffold loaded with cannabidiol (CBD) and found that several MSCs migrated into the rat radial bone defect site (Kamali et al., 2019). Li et al. co-transplanted nano-HAp/polyurethane scaffolds with osteoblasts induced by rat-derived MSCs. The results showed that alkaline phosphatase (ALP), Col-I, osteocalcin (OCN), MSX2, and Runt-related transcription factor 2 (RUNX2) increased greatly (Li L. et al., 2019). Aslam et al. developed a tri-composite biomaterial with marine-derived chitosan, fucoidan, and HA, loaded with MSCs, showing prospects for fracture treatment, promoting cell proliferation and osteogenesis (Aslam et al., 2023). Zarei et al. reported the fabrication of poly (lactic acid)/Ti6Al4V@calcium phosphate core-shell nanocomposite scaffolds through fused deposition modeling (FDM). These scaffolds demonstrated enhanced mechanical properties and *in vitro* biocompatibility, supporting the attachment, differentiation, and proliferation of human AD-MSCs, making them a promising candidate for repairing critical-size bone defects (Zarei et al., 2023). Furthermore, combining bioactive materials and pro-osteogenic factors from MSCs also has a positive effect on bone regeneration. BMPs belong to the TGF-β family, which are particularly important in bone and cartilage formation, as they stimulate the proliferation and differentiation of osteoblasts (Mengrui et al., 2024). Sun et al. installed a recombinant adenovirus with BMP-2 into a hydrogel scaffold, and clear bone formation was observed after 6 weeks in a mouse model (Sun et al., 2020). The secretome of MSCs overexpressing BMP-9 enhanced the osteoblast differentiation and bone repair capabilities of MSC by upregulating RUNX2, ALP and osteopontin protein expression (Calixto et al., 2023). Schoonraad et al. used a matrix-metalloproteinase (MMP)-sensitive hydrogel nanocomposite, comprised of poly(ethylene glycol) crosslinked with MMP-sensitive peptides, tethered RGD, and entrapped HA nanoparticles, proving that rhBMP-2 and rhBMP-9 enhance the osteogenic potential of human MSC embedded in hydrogels (Schoonraad et al., 2023). He et al. developed an acoustically responsive biomimetic hydrogel scaffold complex that releases SDF-1/BMP-2 cytokines via pulsed ultrasound to promote the endogenous BMSCs recruiting and bone regeneration (He et al., 2023). These studies demonstrate that MSCs are currently a well-established option in the field of bone tissue engineering and regenerative medicine. However, their availability and capacity for self-renewal remain constrained in practice. Recent advances in somatic cell reprogramming offer a promising solution to these challenges (Takahashi and Yamanaka, 2006). The emergence of iPSCs has revolutionized the field of regenerative medicine, particularly in the realm of bone healing and regeneration. As researchers explore their capabilities, studies have confirmed that iPSC-derived MSCs demonstrate superior effectiveness in regenerating nonunion bone defects in murine models compared to traditional BM-MSCs (Sheyn et al., 2016). Transplantation of iPSC-seeded PLGA/aCaP scaffolds may improve bone regeneration in critical-size bone defects in mice (Kessler et al., 2024). Further research has demonstrated that iPSCs can generate high yields of osteogenic cell-matrix (OCM) *in vitro*, with osteogenic activity surpassing that of BMP-2. This development offers a biologically active and scalable biomaterial strategy without donor cells for enhancing bone regeneration in patients with delayed or failed bone healing. This donor-free cell products mitigates concerns related to donor variability and presents a higher safety profile compared to synthetic biomaterials (McNeill et al., 2020; Hanetseder et al., 2023). Therefore, the extending scope of bioactive material selection and the applications of various composite factors will lead to important developments and improvements in bone defect therapy soon.

Moreover, significant effectiveness of pure MSCs or the combination of MSCs and biological scaffolds has been shown in clinical trials. A systematic review and meta-analysis confirmed that MSCs therapy significantly augmented the progress of bone regeneration and significantly reduced the incidence of poor recovery (Yi et al., 2022). For example, scholars combined MSCs loaded on fibrin clots and implanted them into an upper bone nonunion. The remodeling rate of bone was significantly enhanced (Giannotti et al., 2013). Hernigou et al. implanted BM-MSCs from the posterior iliac ridge at the contact site between the acetabular graft and the host and observed a rapid increase in graft-host bone osseointegration (Hernigou et al., 2014). In tibial plateau collapse, the application of autologous MSCs/β-tricalcium phosphate (β-TCP) increased the bone regeneration ability and promoted patient rehabilitation (Gan et al., 2008; Chu et al., 2019). Additionally, MSC exosomes integrated into the β-TCP scaffold increased the osteogenic activity in bone defect sites (Zhang et al., 2016). The combination of amplified hBM-MSCs with biomaterials can effectively obtain bone consolidation (Gómez-Barrena et al., 2020). The combination treatment of WJ-MSCs and teriparatide has been shown to be feasible and tolerable in Phase I/IIa studies, and has a clinical benefit for fracture healing by promoting bone architecture (Shim et al., 2021). A case report has shown that the implantation of MSCs and secretome to treat osteoporotic compression fractures can improve the quality of bones and surrounding tissues (Rahyussalim et al., 2023). In short, patients with fractures benefit from MSCs administration. These findings demonstrate the clinical feasibility of combining MSCs with bioactive materials and are potentially useful drugs for bone regeneration. While these clinical applications have achieved satisfactory results, safety concerns and the proper screening needed have made determining how to accurately and suitably enrich cells in scaffolds and should be considered in bone regeneration.

### 4.2 MSCs in pathological bone defects

Pathological bone defects mainly refer to bone loss caused by congenital disease, deformity, bone tumors, osteonecrosis, osteogenic dysfunction, etc., These defects are typically characterized by a long cycle of slow repair and long-term nonhealing (Arrigoni et al., 2017). There remains an enormous need for effective strategies to repair pathological bone defects. Therefore, it is of great clinical value to study the application of MSCs in pathological defects.

#### 4.2.1 Tumor defects

Bone tumors, especially those with malignant potential, frequently occur around the knee joint in the skeletal system. Osteosarcoma is one of the most prevalent malignant bone tumors, characterized by its rapid growth and high tendency for metastasis. (Shoaib et al., 2022). Surgery is a common treatment method; however, it often results in the retention of very large bone defect (Han et al., 2021; Machoň et al., 2022). Therefore, finding effective strategies to promote bone regeneration in bone tumors post operation remains a challenge for clinical experts. As pluripotent stem cells located in connective tissue, modified MSCs have the capability to not only promote osteogenesis and facilitate defect repair, but they also function as effective transport carriers for therapeutic agents. Additionally, modified MSCs exhibit antitumor properties by targeting and inhibiting tumor growth, offering a potential dual role in both regenerative medicine and cancer therapy (Qiao et al., 2015).

Osteolysis is a common pathological change of all osteosarcomas. It is a process of abnormal changes in physiological bone reconstruction and excessive bone resorption (deSantos and Edeiken, 1982; Julie et al., 2011). Nuclear factor κB receptor activator of nuclear factor ligand κB ligand (RANKL)/nuclear factor κB receptor activator of nuclear factor ligand κB (RANK)/osteoprotegerin (OPG) is a key pathway in the regulation of bone injury and osteolysis (Zhang et al., 2022). This pathway is important not only for bone homeostasis maintenance but also for pathological regulation. Previous studies found that increasing OPG expression could reduce tumor-induced osteolysis by inhibiting osteoclasts and weakening osteosarcoma (Lamoureux et al., 2007). Since transgenic MSCs can introduce antitumor factors into tumor sites, Qiao et al. overexpressed the OPG gene in MSCs and then applied them due to the aggregation of fluorescently labeled MSCs-OPG to osteosarcoma nude mice. The results showed that MSCs-OPG play an inhibitory role in osteosarcoma proliferation and osteolysis (Qiao et al., 2015).

MSCs could also serve as a promising platform for the targeted delivery of therapeutic nanomedicines. These medicines have the potential to regulate local immunity, enhance the osteogenic differentiation of MSCs and increase their immunomodulatory effects (Chang et al., 2021). This combination may yield a multifaceted approach to combating osteosarcoma. For example, aclitaxel on MSCs was accurately transported to the tumor site by MSC homing, showing a dose-dependent antitumor effect *in vitro* (Hu et al., 2022). Kalia et al. observed that when a HAp-coated circle was transplanted with autologous MSCs into a sheep bone tumor model, bone growth increased significantly, especially in the area near the coating circle, compared with the control group (Kalia et al., 2006). Lenna and workers performed photodynamic therapy (PDT) with MSCs loaded with AlPcS4@FNPs *in vitro* and *in vivo*, where AlPcS4 is a photosensitizer that can be activated with an LED source, and showed that MSCs can act as carriers to inhibit osteosarcoma growth (Lenna et al., 2020). In similar studies, it was found that MSCs loaded with a photosensitizer (TPPS@FNPs) could promote the release of ROS from osteosarcoma cells (Duchi et al., 2013). Suitable ROS stimulus can further induce macrophages polarization from M1 to M2 phenotype, which contributes to bone formation around implants (Tsai et al., 2021). Additionally, exosomes derived from MSCs also have therapeutic value (Nakao et al., 2021). Abello and colleagues found that labeled exosomes from human UC-MSCs could accumulate in the tumor site for more than 24–48 h after injection, suggesting that exosomes may be a prospective substance to treat bone tumors due to their tumor-targeting features (Abello et al., 2019).

However, it is unfortunate that some studies have also found that MSCs may promote osteosarcoma. For instance, a study by Avnet et al., MSCs were co-injected with osteosarcoma cells into subcutaneous and orthotopic models demonstrated that lung metastasis significantly increased because of the acidosis induced by MSCs (Avnet et al., 2021). Compared with osteoclast precursors, MSCs accelerated the local proliferation of osteosarcoma cells, but did not intensify the process of osteolysis and metastasis (Pierre et al., 2015). Moreover, exosomes derived from MSCs with overexpressed miR-21-5p can enhance the proliferation and invasion of osteosarcoma cells by activating the PI3K/Akt/mTOR signaling pathway, both *in vitro* and *in vivo* (Qi et al., 2021). It is astonished that MSCs could directly transform into Ewing sarcoma cells by engineering the t(11; 22) (q24; q12) translocation together with *CDKN2A* gene mutations (Sole et al., 2021). Thus, contradictory results have been reported, possibly due to nonstandard protocols, variations in tumor types and stages, the absence of specific cell markers for accurate identification, and the susceptibility of MSCs to their surrounding microenvironment. Consequently, a comprehensive analysis of the effects and underlying mechanisms of MSCs in bone tumors is warranted for future research.

#### 4.2.2 Osteogenic dysfunction

Primary osteogenic dysfunction is often caused by inherited metabolic diseases. Osteogenesis imperfecta (OI), known as both brittle bone disease and china doll, is an autosomal inherited disorder that mainly manifests as generalized osteoporosis due to defects in osteogenesis and fibroblast function (Marom et al., 2016). OI-derived iPSCs exhibited decreased cell growth and impaired osteogenic differentiation and collagen expression (Duangchan et al., 2021). Otsuru et al. first reported that the use of allogeneic MSC transplantation in children with OI resulted in significant osteogenesis in osteoporosis locations (Otsuru et al., 2012). Furthermore, MSCs was injected into fetuses with OI during pregnancy and found significant improvements in bone line growth, reduced fracture incidence, and improved growth trajectory *postpartum* (Götherström et al., 2014). In the TERCELOI clinical trial, reiterative infusions of histocompatible MSCs in pediatric OI patients were safe, improved bone parameters, and elicited a pro-osteogenic paracrine response (Infante et al., 2021). It was observed that the expression of the TGF-β pathway was enhanced in the serum of the most severe pediatric patients with OI, which were modulated after MSCs therapy (Infante et al., 2022). Hypophosphatasia (HPP) is also a hereditary disease characterized by incomplete mineralization of bones and teeth (Khan et al., 2024). Clinical studies have reported that allogeneic MSCs injected into HPP patients significantly strengthened bone density (Taketani et al., 2015). Allogeneic MSC transplantation, along with the local implantation of osteogenic constructs incorporating MSCs and porous hydroxyapatite ceramics, demonstrated consistent improvement in respiratory and skeletal abnormalities in a patient with perinatal HPP (Tadokoro et al., 2009). Congenital pseudarthrosis of the tibia (CPT) is a rare orthopedic disorder characterized by a spontaneous nonhealing fracture, often associated with neurofibromatosis type I (NF1) (Van Royen et al., 2016, p. 1). Granchi et al. reported an effective achievement in bone tissue regeneration by using a regenerative strategy involving MSCs combined with platelet-rich fibrin (PRF) as a source of growth factors (Granchi et al., 2012). Moreover, the effectiveness of *in situ* injection of bone marrow aspirate concentrate (BMAC), as an adjunctive treatment for CPT, and autologous MSC injection combined with external fixation at the pseudarthrosis site resulted in faster bone healing in comparison to external fixation (Memeo et al., 2020). Kurniawan et al. explored the efficacy of combining UC-MSCs with secretome in treating CPT, revealing favorable outcomes such as high primary union rates and significant functional improvement in their case series (Kurniawan et al., 2023). Therefore, using MSC-based therapies might be a dependable way to treat both acquired and inherited bone defects. Advances in this field could lead to more effective treatments, potentially transforming the management of complex bone disorders and improving patient outcomes significantly.

#### 4.2.3 Osteonecrosis

Osteonecrosis is typically caused by the lack of blood supply to bone tissue, leading to a pathological state characterized by cell death. This condition is often secondary to the effects of medications, radiation, or other chronic diseases (Shah et al., 2015). Jiang et al. investigated the efficacy of MSCs in a mouse model of glucocorticoid-induced osteonecrosis. They combined MSCs with Rab001, along with an MSC bone-targeted agent, and showed that this combination effectively directs MSCs to trabecular and endocortical bone surfaces and significantly inhibits the progression of osteonecrosis (Jiang et al., 2021). Additionally, the intravenous injection of MSCs into rabbits with hormonal femoral head necrosis resulted in an increased concentration of MSCs around the femoral blood vessels after 3 days and effectively alleviated the severity of osteonecrosis (Ueda et al., 2017). In several clinical trials with long-term follow-ups, the injection of autologous BM-MSCs into patients with femoral head necrosis significantly reduced the rate of artificial hip replacement and improved disease outcome compared to simple core decompression without cellular intervention (Daltro et al., 2015; Hernigou et al., 2018; Hernigou et al., 2021; Kang et al., 2018; Gómez-Barrena et al., 2021; Diri et al., 2023; Ulusoy et al., 2023). Recent studies emphasize the promising application of iPSCs in treating osteonecrosis of the femoral head through various mechanisms. iPSC-derived MSCs exhibit robust regenerative potential, promoting bone repair and angiogenesis in necrotic areas (Zhou et al., 2021). Additionally, exosomes secreted by human-iPSC-MSCs (hiPS-MSC-Exos) have demonstrated their potential as a therapeutic strategy for osteonecrosis of the femoral head by enhancing local angiogenesis and preventing bone loss through the activation of the PI3K/Akt signaling pathway in endothelial cells (Liu et al., 2017). Combining hiPS-MSC-Exos with miR-135b has been shown to further alleviate bone loss and promote osteoblast proliferation while inhibiting apoptosis (Xiang et al., 2020). These advancements underscore the transformative potential of MSCs therapies, particularly iPSCs, in not only halting the progression of osteonecrosis but also in fostering significant improvements in bone regeneration and overall patient outcomes.

In studies examining medication-related osteonecrosis of the jaw (MRONJ), the effects observed following treatment with MSCs were comparable to those found in therapies for hormone-related femoral head necrosis. For instance, Kaibuchi et al. observed that MSCs aggregated around new blood vessels after injecting MSCs labeled with fluorescent dyes into a rats model of MRONJ (Kaibuchi et al., 2016). While diseased MSCs from mice with MRONJ displayed inferior characteristics compared to MSCs from untreated mice, the exchange of cellular contents between control MSCs and diseased MSCs significantly contributed to the treatment potential of diseased MSCs (Matsuura et al., 2016). Moreover, the addition of CM from cultured MSCs *in vitro* to the model rats above promoted the migration, proliferation, and osteogenic differentiation of MSCs at osteonecrosis sites (Ogata et al., 2017; Ogata et al., 2018). Allogeneic BM-MSCs in extractions sites ameliorates MRONJ incidence in zoledronic acid-treated rats compared to non-MSC treatments (Rodríguez-Lozano et al., 2020). These patients with MRONJ exhibited varying degrees of pathological improvement. In addition to etiology-specific treatments, the use of MSCs has shown significant clinical outcomes for patients with bone necrosis. However, the detailed immune regulatory mechanisms of MSCs in treating osteonecrosis, as well as the interactions between allogeneic MSCs and various cell populations in the pathological microenvironment, need to be further elucidated.

## 5 Conclusion

Repairing bone defects caused by trauma, surgery, tumors, hypoplasia, and other factors has long posed a significant challenge in the field of bone tissue engineering. MSCs, a major type of adult stem cells, can be acquired from various tissues and exhibit low immunogenicity, multidirectional differentiation potential, and excellent compatibility and stability, making them ideal candidates for tissue repair and immune regulation. Currently, MSCs are drawing increasing attention for the treatment of bone defects. The regulated mechanisms underlying their bioactive behavior have been elucidated, revealing that these mechanisms involve differentiation, immune regulation, and paracrine effects. In this review, we primarily discussed the present application status of MSCs, emphasizing their regulatory effect in bone defects repair. MSCs affect osteogenesis through three-dimensional interactions with other cells, particularly macrophages. In summary, the applications of MSCs can be categorized into three aspects. First, there is the direct application of MSCs in bone healing, which remains the most common clinical treatment method. It also includes the use of MSCs as effective cells combined with bioactive materials, which can be applied via local or intravenous injection. Such approaches not only improve the biocompatibility of biomaterials but also improve the loading capacity and targeting of MSCs. Second, MSC-CM plays a crucial role by containing cytokines that activate specific signaling pathways to maintain balance in the bone microenvironment. Additionally, exosomes derived from MSCs also represent an important therapeutic component for bone defects because they carry multiple active factors, including DNA, RNA, and proteins. Third, MSCs can serve as vehicles or be modified into a specific complex to deliver one or more agents to the bone defect site through MSC homing. While MSCs have broad prospects in clinical bone defect therapy, further studies are necessary to investigate the underlying molecular mechanisms, their secretory factors and their influence on bone homeostasis and regeneration. It is imperative to expedite the development of combination therapies involving MSCs and biomaterials to accelerate their clinical translation. However, in extracting the clinical value of MSCs, it is equally necessary to recognize the importance of addressing regulatory considerations and potential obstacles, such as long-term safety and effectiveness.
